# Incidence Rates and Time Trends of Pancreatic Cancer in the Golestan Province, Northeastern Iran, 2006-2019

**DOI:** 10.34172/aim.31168

**Published:** 2024-09-01

**Authors:** Sajjad Kaabe, Taghi Amiriani, Mehrdad Teimoorian, Sima Besharat, Faezeh Salamat, Susan Hasanpour-Heidari, SeyyedMehdi Sedaghat, Hamideh Sadeghzadeh, Gholamreza Roshandel

**Affiliations:** ^1^Golestan Research Center of Gastroenterology and Hepatology, Golestan University of Medical Sciences, Gorgan, Iran; ^2^Stem Cell Research Center, Golestan University of Medical Sciences, Gorgan, Iran; ^3^Clinical Research Development Unit (CRDU), Sayad Shirazi Hospital, Golestan University of Medical Sciences, Gorgan, Iran; ^4^Deputy of Public Health, Golestan University of Medical Sciences, Gorgan, Iran

**Keywords:** Golestan province, Incidence, Pancreatic cancer, Temporal variation

## Abstract

**Background:**

Pancreatic cancer (PC) is one of the most malignant cancers with a poor prognosis. Despite advances in the diagnosis and management of PC, the survival rate remains low. In Iran, the incidence of PC is increasing, with mortality rates nearly doubling over the past 25 years. Therefore, this study was designed to assess the temporal variations and incidence of PC in Golestan province, as a prominent hub for gastrointestinal cancers in Iran.

**Methods:**

In this cross-sectional study, patient information was obtained from the Golestan Population-Based Cancer Registry (GPCR) from 2006 to 2019. We calculated age-standardized incidence rates (ASRs) using the World standard population and reported the rates per 100000 persons-year. To compare ASRs across sexes and residence areas, incidence rate ratios (IRR) were calculated using Poisson regression models. We calculated the estimated annual percentage changes (EAPC) to assess time trends in incidence rates of PC in Golestan during the study period.

**Results:**

Among a total of 560 PC new cases (mean age of 63.72 years), 46.61% were diagnosed through clinical or paraclinical methods. The crude incidence rate and ASR were 2.24 and 2.95 (95% CI: 2.70‒3.20) per 100000 persons-year, respectively. The ASR of PC was significantly higher in males (3.78; 95% CI: 3.37‒4.19) than females (2.17; 95% CI: 1.88‒2.46) (IRR=1.71; *P*<0.01). The ASR was higher in the urban (3.23; 95% CI: 2.88‒3.58) compared to the rural population (2.65; 95% CI: 2.30‒3.00) (IRR=1.23; *P*=0.02). The ASR of PC increased from 1.97 to 3.53 during 2006 to 2019 with an EAPC of 4.39 (95% CI: -3.56 to 12.75). The EAPCs were 4.85% and 4.37% in women and men, respectively.

**Conclusion:**

Our study showed that the incidence of PC is increasing in the Golestan province. Also, the incidence rate was higher in men, elderly people, and the urban population.

## Introduction

 Pancreatic cancer (PC) is a type of cancer that ranks as the 12th most common form of cancer globally. In 2020, an estimated 495 773 individuals were diagnosed with this disease. Unfortunately, it is also the 7th deadliest cancer in the world, with 466 000 deaths reported.^1^ PC is considered to be one of the most lethal forms of cancer. Despite advancement in diagnosis and treatment, the prognosis for patients remains poor, with a 5-year survival rate of only 9%. Over the past decade, the incidence and mortality rate of PC have been on the rise worldwide, regardless of gender.^2^ Projections based on demographic shifts suggests that by the year 2040, PC will become the second leading cause of cancer-related deaths in the United States.^3^ This underscores the urgent need for increased research, prevention strategies, and improved treatment options to combat this devastating disease.

 In 2020, the global age-standardized incidence rate (ASR) of PC was 4.9 per 100 000 individuals. Men had a slightly higher ASR of 5.7 per 100 000 people, while women had a lower rate of 4.1 per 100 000 people. Hungary, Uruguay and Japan were identified as having the highest ASRs of PC, with ASRs of 11.2, 10.7 and 9.9 per 100 000 people, respectively. The ASR of Iran in 2020 was 3.8 per 100 000 people, with a rate of 4.7 per 100 000 people for men and 2.9 per 100 000 people for women.^4^ Over the past three decades, Iran has seen a rise in PC incidence, with the mortality rate nearly doubling during this time period.^5^

 The exact causes of PC are not fully understood; however, certain factors like age, gender (being male), smoking, alcohol consumption, obesity, diabetes, specific dietary habits, family history, and genetic mutations are known to significantly increase the risk of developing the disease.^6,7^

 Over the past few years, there has been a noticeable increase in the number of PC cases in wealthier countries. These countries, known for their higher levels of income and better living conditions, have seen a rise in the number of elderly individuals, leading to an increased risk of PC. Additionally, these nations have better access to healthcare services, advanced diagnostic tools, and improved cancer registration systems, resulting in identifying more cases of the disease. Moreover, lifestyle changes have also played a crucial role in exposing more people to risk factors associated with PC, thus contributing to the overall increase in its incidence.^8^

 PC presents with vague symptoms, which can make it difficult to diagnose early. Most patients are unfortunately diagnosed when the cancer has already spread to other parts of the body, with only 10% of them found in their initial stages.^9^ This highlights the aggressive nature of PC, which often leads to a very poor prognosis, due to limited treatment options once the disease has progressed. Unfortunately, only about 20% of newly diagnosed patients are suitable candidates for surgery.^10^

 The trend of PC has been increasing in Iran.^11-13^ Considering the fact that the Golestan province has been identified as a prominent area for gastrointestinal cancers in Iran, this increasing trend can be expected in this geographical region. Also, analyzing epidemiological data can offer valuable perspectives in gaining a better understanding of PC.^14-15^ In this study, we aimed to investigate the incidence and epidemiological profile of PC in the Golestan province during the designated study period.

## Materials and Methods

 In this cross-sectional study, all individuals who were diagnosed with primary PC between 2006 and 2019 were included. Those with benign tumors as well as those with recurrent PC were excluded. Data regarding patients with PC was retrieved from the Golestan Population-based Cancer Registry (GPCR) database. The GPCR is a high-quality cancer registry and a voting member of the International Association of Cancer Registries (IACR). The GPCR uses the internationally acceptable standards of cancer registration, developed by the International Agency for Research on Cancer (IARC). According to these standards, the GPCR uses a data extraction form consisting of patients’ data (first name, last name, identification number, father’s name, sex, age, and residence area) and data on tumor characteristics (topography, morphology, behavior and diagnosis method). The registry utilizes various methods such as histopathology, clinical or paraclinical approaches, and death certificate only (DCO) methods to document the diagnosis of PC. The GPCR uses different indices for controlling the quality of data, including the percentage of cases with microscopic verification (MV%). Patient data was encoded based on the international ICD-O3 coding system and entered into the CanReg software.

 In this study, patients with specific codes (C250-C259) were included in the study. Then, using this data, the crude incidence rate was calculated. We used the 18-group World Standard Population (0‒4, 5‒9, …, ≥ 85) for standardization of incidence rates.^16,17^ All rates are reported per 100 000 person-years. Poisson regression models were used to compare the incidence rates across genders and places of residence. Incidence rate ratios (IRR) with 95% confidence intervals (CI) were calculated. *P* values less than 0.05 were considered as statistically significant. The estimated annual percentage change (EAPC) with 95% CI were calculated to assess time trends in incidence rates of PC. The statistical inference for time trend in incidence rate was made by estimated 95% CI of EAPC. If the 95% CI of EAPC does not contain the value 0, it is considered as a statistically significant trend.

## Results

 Overall, 560 new cases of PC were registered in the GPCR during 2006–2019. The mean age of the patients was 63.72 (13.42) years. The majority of the cases were male (62.3%) and living in urban areas (58.40%).

 Two hundred and forty-eight (44.3%) cases were diagnosed by the histopathological method, 261 (46.61%) by clinical or paraclinical methods and the remaining 51 (9.10%) cases were diagnosed by the DCO method.

 The ASR of PC was 2.95 per 100 000 person-years (95% CI: 2.70–3.20). The ASR of PC was significantly higher in males (ASR = 3.78; 95% CI: 3.37–4.19) than females (ASR = 2.17; 95% CI: 1.88–2.46) (IRR = 1.71; 95% CI: 1.44–2.03; *P* < 0.01). We also found a considerably higher ASR for PC in the urban (ASR = 3.23; 95% CI: 2.88–3.58) than the rural population (ASR = 2.65; 95% CI: 2.30–3.00) (IRR = 1.23; 95% CI: 1.04–1.46; *P*= 0.02) (Table 1).

**Table 1:** Number, Crude Rate, ASR and 95% CI of ASR (Per 100 000 Person-Years) for Pancreatic Cancer in Golestan, Iran During 2006‒2019

	**Number**	**Crude Rate**	**ASR**	**ASR-L**	**ASR-U**
Total population	560	2.24	2.95	2.70	3.20
By gender					
Male	349	2.78	3.78	3.37	4.19
Female	211	1.69	2.17	1.88	2.46
By residence area					
Urban	327	2.52	3.23	2.88	3.58
Rural	233	1.93	2.65	2.30	3.00

ASR-L, Lower CI limit for ASR; ASR-U, Upper CI limit for ASR.

**Figure 1:**
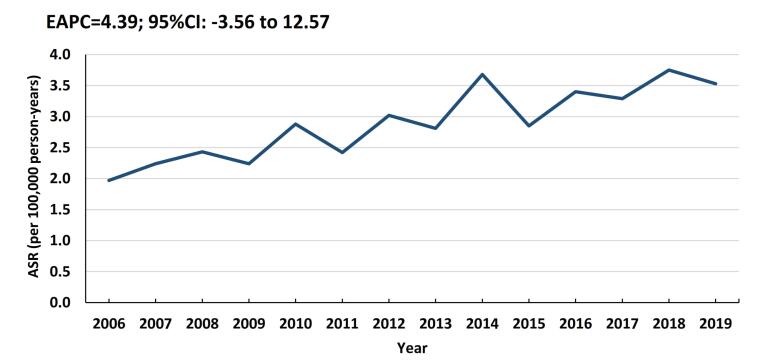
Time Trends of Age-Standardized Incidence Rate (ASR) (Per 100 000 Person-Years) of pancreatic cancer in Golestan, Iran, 2006‒2019. EAPC, estimated annual percentage change.

 There were no significant trends in the incidence rates of PC in our population (EAPC = 4.39; 95% CI: -3.56 to 12.75) (Figure 1).

**Figure 2:**
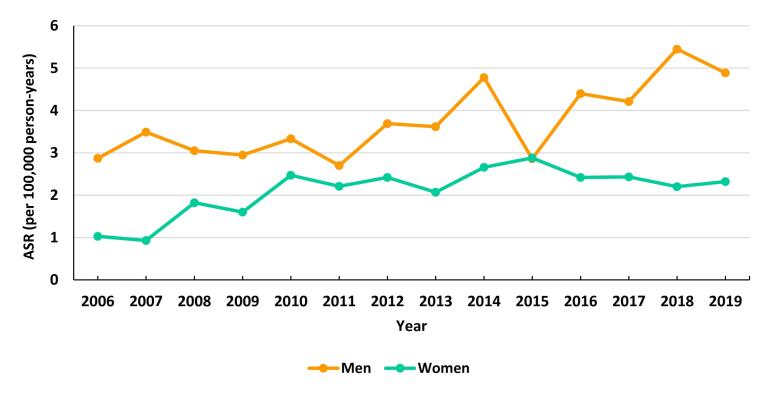
Time Trends of Age-Standardized Incidence Rate (ASR) (Per 100 000 Person-Years) of Pancreatic Cancers by Gender

 As shown in Figure 2, our results showed no significant time trends for the ASR of PC in males (EAPC = 4.37; 95% CI: -2.48 to 11.69) and females (EAPC = 4.85; 95% CI: -4.23 to 14.79).

**Figure 3:**
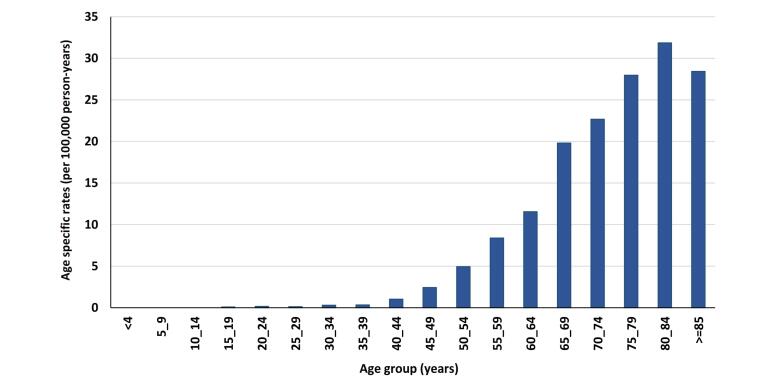
Age-Specific Incidence Rate (ASR) (Per 100 000 Person-Years) for Pancreatic Cancer in Golestan, Iran, 2006‒2019

 The age-specific incidence rate for PC in the total population was highest in the age group of 80‒84 years (Figure 3). PC was not detected in the younger male population, i.e. those aged 14 and younger. In residents of urban and rural areas, the age-specific incidence rate was highest in the elderly population.

**Figure 4:**
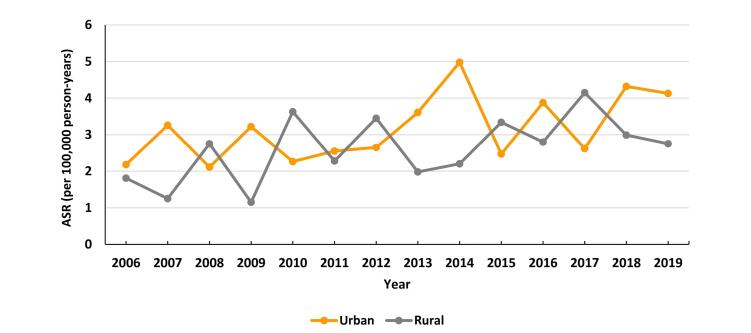
Time Trends of Age-Standardized Incidence Rate (ASR) (Per 100 000 Person-Years) of Pancreatic Cancer by Area of Residence

 We did not find significant temporal changes in the incidence rates of PC in the urban (EAPC = 3.96; 95% CI: -3.42 to 11.90) and rural populations (EAPC = 4.54; 95% CI: -3.62 to 13.38) (Figure 4).

**Figure 5:**
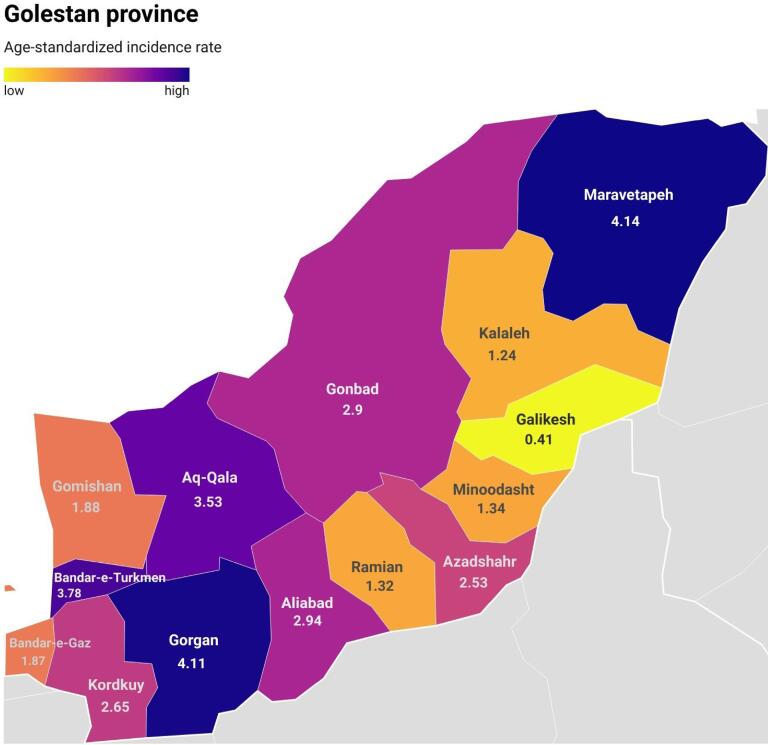
Geographical Distribution of the Age-Standardized Incidence Rate (Per 100 000 Person-Year) of Pancreatic Cancer in the Golestan Province, Iran, 2006‒2019, by City

 The number, crude rate, ASR and 95% CI of ASR for PC by city are presented in Table 2. The rate of PC was highest in the most Turkmen-populated cities of the provinces Maravetapeh, Bandar-e-Turkmen and Aq-Qala. (Figure 5)

**Table 2:** Number, Crude Rate, ASR and 95% CI of ASR (Per 100 000 Person-Years) for Pancreatic Cancer in Golestan, Iran, 2006‒2019, by City

	**Number**	**Crude Rate**	**ASR**	**ASR-L**	**ASR-U**
Aliabad	40	2.11	2.94	2.00	3.88
Aq-qala	40	2.28	3.53	2.39	4.67
Azadshahr	26	1.98	2.53	1.53	3.53
Bandar-e-gaz	14	2.16	1.87	0.85	2.89
Bandar-e-turkmen	46	2.74	3.78	2.66	4.90
Galikesh	1	0.40	0.41	0.00	1.21
Gomishan	4	1.39	1.88	0.02	3.74
Gonbad	95	2.06	2.9	2.29	3.51
Gorgan	215	3.35	4.11	3.54	4.68
Kalaleh	18	0.85	1.24	0.63	1.85
Kordkuy	28	2.85	2.65	1.63	3.67
Maravetapeh	4	1.64	4.14	0.00	8.35
Minoodasht	17	1.04	1.34	0.69	1.99
Ramian	12	1.01	1.32	0.56	2.08

ASR-L: Lower CI limit for ASR; ASR-U: Upper CI limit for ASR

Tables 3 and 4 show the number, crude rate, ASR, and 95% CI of ASR for PC by city, sex, and residence area. Table 5 shows the number, crude rate, ASR, and 95% CI of ASR for PC by sex and year.

**Table 3:** Number, Crude Rate, ASR and 95% CI of ASR (Per 100 000 Person-Years) for Pancreatic Cancer in Golestan, Iran, 2006‒2019, by City and Sex

	**Male**					**Female**				
	**Number**	**Crude Rate**	**ASR**	**ASR-L**	**ASR-U**	**Number**	**Crude Rate**	**ASR**	**ASR-L**	**ASR-U**
Aliabad	28	2.96	4.49	2.78	6.20	12	1.27	1.56	0.66	2.46
Aq-qala	20	2.29	3.80	2.06	5.54	20	2.26	3.36	1.85	4.87
Azadshahr	20	3.05	3.82	2.10	5.54	6	0.91	1.29	0.25	2.33
Bandar-e-gaz	9	2.80	2.37	0.76	3.98	5	1.54	1.35	0.13	2.57
Bandar-e-turkmen	28	3.34	4.83	2.99	6.67	18	2.14	2.85	1.52	4.18
Galikesh	1	0.78	0.80	0.00	2.37	0	0.00	0.00	0.00	0.00
Gomishan	2	1.40	2.47	0.00	5.90	2	1.37	1.51	0.00	3.61
Gonbad	53	2.29	3.24	2.32	4.16	42	1.82	2.56	1.76	3.36
Gorgan	149	4.61	5.75	4.79	6.71	66	2.07	2.50	1.89	3.11
Kalaleh	6	0.57	0.88	0.15	1.61	12	1.12	1.56	0.62	2.50
Kordkuy	16	3.24	3.22	1.57	4.87	12	2.45	2.14	0.91	3.37
Maravetapeh	1	0.81	1.69	0.00	5.00	3	2.46	6.29	0.00	13.60
Minoodasht	9	1.11	1.42	0.46	2.38	8	0.97	1.22	0.36	2.08
Ramian	7	1.18	1.50	0.36	2.64	5	0.84	1.13	0.11	2.15

ASR-L: Lower CI limit for ASR; ASR-U: Upper CI limit for ASR

**Table 4:** Number, Crude Rate, ASR and 95% CI of ASR (Per 100 000 Person-Years) for Pancreatic Cancer in Golestan, Iran, 2006-2019, by City and Residence Area

	**Urban**					**Rural**				
	**Number**	**Crude Rate**	**ASR**	**ASR-L**	**ASR-U**	**Number**	**Crude Rate**	**ASR**	**ASR-L**	**ASR-U**
Aliabad	21	2.09	2.78	1.55	4.01	19	2.14	3.12	1.69	4.55
Aq-qala	7	1.28	1.61	0.34	2.88	33	2.73	4.27	2.78	5.76
Azadshahr	12	1.64	1.93	0.79	3.07	14	2.40	3.34	1.58	5.10
Bandar-e-gaz	10	2.69	2.52	0.91	4.13	4	1.46	1.11	0.00	2.23
Bandar-e-turkmen	31	2.99	4.20	2.69	5.71	15	2.33	3.15	1.52	4.78
Galikesh	0	0.00	0.00	0.00	0.00	1	0.65	0.67	0.00	1.98
Gomishan	2	1.34	2.03	0.00	4.83	2	1.44	1.81	0.00	4.36
Gonbad	45	2.18	2.58	1.80	3.36	50	1.96	3.21	2.29	4.13
Gorgan	166	3.48	4.43	3.74	5.12	49	2.99	3.34	2.38	4.30
Kalaleh	3	0.53	0.64	0.00	1.37	15	0.96	1.46	0.68	2.24
Kordkuy	16	3.07	3.23	1.60	4.86	12	2.59	2.09	0.86	3.32
Maravetapeh	0	0.00	0.00	0.00	0.00	4	1.93	4.75	0.00	9.57
Minoodasht	10	1.62	2.39	0.86	3.92	7	0.68	0.81	0.20	1.42
Ramian	4	0.85	0.99	0.00	2.01	8	1.11	1.56	0.44	2.68

ASR-L: Lower CI limit for ASR; ASR-U: Upper CI limit for ASR

**Table 5:** Number, Crude Rate, ASR and 95% CI of ASR (Per 100 000 Person-Years) for Pancreatic Cancer in Golestan, Iran, 2006‒2019, by Sex and Year

	**Male**					**Female**					**Total**				
**Year**	**Number**	**Crude Rate**	**ASR**	**ASR-L**	**ASR-U**	**Number**	**Crude Rate**	**ASR**	**ASR-L**	**ASR-U**	**Number**	**Crude Rate**	**ASR**	**ASR-L**	**ASR-U**
2006	16	1.99	2.87	1.4	4.34	5	0.62	1.03	0.11	1.95	21	1.3	1.97	1.09	2.85
2007	21	2.56	3.49	1.94	5.04	6	0.72	0.93	0.17	1.69	27	1.64	2.24	1.38	3.1
2008	17	2.03	3.05	1.54	4.56	11	1.3	1.82	0.7	2.94	28	1.67	2.43	1.49	3.37
2009	17	1.99	2.95	1.5	4.4	11	1.28	1.6	0.62	2.58	28	1.64	2.24	1.38	3.1
2010	20	2.3	3.33	1.82	4.84	15	1.72	2.47	1.18	3.76	35	2.01	2.88	1.9	3.86
2011	17	1.92	2.7	1.37	4.03	16	1.8	2.21	1.09	3.33	33	1.86	2.42	1.56	3.28
2012	23	2.57	3.69	2.12	5.26	17	1.89	2.42	1.24	3.6	40	2.23	3.02	2.06	3.98
2013	24	2.65	3.62	2.11	5.13	15	1.66	2.07	0.99	3.15	39	2.15	2.81	1.91	3.71
2014	31	3.38	4.78	3.04	6.52	19	2.08	2.66	1.44	3.88	50	2.73	3.68	2.64	4.72
2015	21	2.26	2.86	1.59	4.13	22	2.39	2.88	1.66	4.1	43	2.32	2.85	1.97	3.73
2016	32	3.41	4.4	2.81	5.99	18	1.93	2.42	1.28	3.56	50	2.68	3.4	2.44	4.36
2017	32	3.37	4.21	2.72	5.7	21	2.24	2.43	1.37	3.49	53	2.81	3.29	2.39	4.19
2018	41	4.27	5.45	3.73	7.17	17	1.8	2.2	1.14	3.26	58	3.04	3.75	2.77	4.73
2019	37	3.82	4.89	3.26	6.52	18	1.88	2.32	1.24	3.4	55	2.86	3.53	2.57	4.49

ASR-L: Lower CI limit for ASR; ASR-U: Upper CI limit for ASR

## Discussion

 The objective of this research was to analyze the trends and prevalence of PC in the Golestan province between the years 2006 and 2019. Our results revealed a lower ASR compared to the national and international rates. The ASR in the Golestan province was found to be lower than neighboring countries such as Armenia (8.9) and Turkey (7.9), but higher than Iraq (2.90), Afghanistan (2.1) and Turkmenistan (2.7).^18^ Additionally, there has been a noticeable increase in ASR in countries like Iran, Saudi Arabia, China and the United States, although the rise is not considered significant.^10-13,19-21^ PC rates are higher in developed countries with higher socio-economic status than developing countries globally. In Iran, provinces with higher socio-economic status have higher cancer incidence rates.^22^ This could be attributed to the availability of advanced diagnostic tools such as CT-scan, ERCP and MRI, as well as better healthcare resources. Additionally, the aging population and changing life styles may also contribute to the higher incidence of PC in these regions.^23,24^

 Incidence rate is considered as an indicator of the mortality of PC, given its poor survival rate. It is anticipated that PC will overcome breast and colorectal cancers in the coming years, becoming the leading cause of death from gastrointestinal cancer by 2040 in America and by 2030 in Germany. Additionally, predictive models show a rising trend in both incidence and mortality rates of PC in Iran, highlighting the urgent need for swift action by healthcare policymakers.^11^

 Within our studied population, the ASR was greater among males compared to females, a trend consistent with findings from prior studies. Although not reaching statistical significance, both genders showed an increasing trend, a pattern also observed in other studies in Iran and other countries.^10-11,13,19-21,25^ The higher rate in the male population may be attributed to risky behaviors such as smoking, alcohol consumption and drug abuse, which are more common in men worldwide. Our study found that the mean annual incidence changes were higher in females compared to males, and the ratio of male to female decreased steadily over time. Alvand et al in Iran (2017) noted that the annual incidence rate change was higher in males (10.47%) than females (8.54%).^11^ These results, along with other research,^20^ indicated a rising trend of obesity and overweight among Iranian women, accompanied by an increase in diabetes. Notably, the rate of increase in these health issues is higher in women compared to men.^26-27^ Moreover, alcohol consumption and the associated disorders are on the rise among women in America. It is suggested that limitations surrounding this issue in Iran could potentially contribute to similar trends.^28^

 As individuals grow older, the rate of PC also tends to increase, as evidenced by our study. The peak age group in our study was 80‒84 years. Studies in different parts of Iran have shown results similar to ours.^11,13^ An epidemiological review in Iran reported that 80% of PC cases were identified between 60 to 80 years of age.^21^ The rise in the elderly population in Iran (people aged 65 and above) from 3.39% in 1991 to 5.72% in 2011^13^ may explain the patterns observed in our study and similar studies in Iran. The same pattern is also seen in some other parts of the world. Similar results have been reported in Saudi Arabia (75 years and older), America (over 60 years) and China (between 85‒89 years).^10,19-20^ As the pancreas ages, it undergoes various pathological changes such as increased fatty tissue replacement, fibrosis, lymphoplasmacytic infiltration, amyloid deposition, and neoplastic alteration. These age-related changes can make a person more vulnerable to pancreatic ductal adenocarcinoma.^29^

 Within our research sample, we observed that the ASR was notably higher in urban regions compared to rural regions. This finding aligns with prior studies conducted by Yin in China, Baum in Egypt, and Segel in the United States.^19,25,30^ The disparity could be attributed to factors such as increased rates of smoking, unhealthy diet characterized by high fat and sugar intake, excessive alcohol consumption, elevated rates of diabetes, lack of physical activity, and sedentary lifestyles prevalent in urban areas. Furthermore, the accessibility of medical care and availability of advanced diagnostic tools in urban settings may contribute to the higher incidence rates observed in these areas.

 The most common method of diagnosing PC in our research was either through clinical or paraclinical methods, followed by histopathology and death certificate data. This finding aligns with Baum’s study in Egypt, but contrasts with the studies by Alvand et al in Iran, Yin in China, and Elamyal in Libya, where histopathology was the preferred diagnostic method.^11,19,25,31^ The challenging aspect of tumor location lies in the difficulty of obtaining tissue samples, particularly in the vulnerable and elderly patients. Additionally, the low survival rate of PC often results in patients being diagnosed at advanced stages leading to death before further diagnostic methods can be utilized.^32^

 Our research revealed that the city of Gorgan, the capital of the Golestan province, along with the cities of Bandar Turkmen, Aq-Qala, and Maraveh Tape, had the highest age-adjusted incidence rates. This can be attributed to the central location of Gorgan city, which houses all diagnostic and treatment facilities as well as a large number of healthcare professionals. The Golestan province is known for its diversity, with immigrants and residents of various races coexisting. The majority of residents in the other mentioned cities are Turkmens. This finding can be attributed to a complex interplay of genetic predisposition and environmental influences.^6^ Lifestyle behaviors such as smoking cigarettes and hookah, using opium and *nass* and poor diets lacking in fresh fruits and vegetables are proven to be key risk factors contributing to the development of PC which are similar to the esophageal cancer risk factors discussed in the Turkmen population in previous studies.^33,34^ Also, there was a significant relationship between esophageal cancer and family history in Turkmens, which shows the influence of genetics.^35^ This may point to the role of some genetics in the development of PC similar to esophageal cancer. Given the lack of similar research on the ethnic diversity of PC in Iran, further epidemiological studies focusing on racial and ethnic disparities are warranted.

## Conclusion

 The standardized incidence rate of PC in our region has been lower than the global and national average. However, there has been a noticeable rise in recent years that cannot be ignored. Factors contributing to this increase may include an aging population, higher prevalence of risk factors, better access to PC screening and diagnosis, and improved cancer reporting systems. As cancer rates are higher among the Turkmen population, it is essential to conduct epidemiological research to explore potential racial disparities within our region and across Iran. It is crucial to address this growing concern and take proactive measures to better understand and manage the rising incidence of PC in our community.
